# Malware Detection for Internet of Things Using One-Class Classification

**DOI:** 10.3390/s24134122

**Published:** 2024-06-25

**Authors:** Tongxin Shi, Roy A. McCann, Ying Huang, Wei Wang, Jun Kong

**Affiliations:** 1 Department of Computer Science, North Dakota State University, Fargo, ND 58102, USA; tongxin.shi@ndsu.edu (T.S.); wei.wang.7@ndsu.edu (W.W.); 2 Department of Electrical Engineering, University of Arkansas, Fayetteville, AR 72701, USA; rmccann@uark.edu; 3 Department of Civil, Construction and Environmental Engineering, North Dakota State University, Fargo, ND 58102, USA

**Keywords:** malware detection, anomaly detection, autoencoder, one-class classification

## Abstract

The increasing usage of interconnected devices within the Internet of Things (IoT) and Industrial IoT (IIoT) has significantly enhanced efficiency and utility in both personal and industrial settings but also heightened cybersecurity vulnerabilities, particularly through IoT malware. This paper explores the use of one-class classification, a method of unsupervised learning, which is especially suitable for unlabeled data, dynamic environments, and malware detection, which is a form of anomaly detection. We introduce the TF-IDF method for transforming nominal features into numerical formats that avoid information loss and manage dimensionality effectively, which is crucial for enhancing pattern recognition when combined with n-grams. Furthermore, we compare the performance of multi-class vs. one-class classification models, including Isolation Forest and deep autoencoder, that are trained with both benign and malicious NetFlow samples vs. trained exclusively on benign NetFlow samples. We achieve 100% recall with precision rates above 80% and 90% across various test datasets using one-class classification. These models show the adaptability of unsupervised learning, especially one-class classification, to the evolving malware threats in the IoT domain, offering insights into enhancing IoT security frameworks and suggesting directions for future research in this critical area.

## 1. Introduction

In the burgeoning field of the Internet of Things (IoT), the increase in interconnected devices has dramatically changed the way people interact with modern technology. It offers marked improvements in efficiency and utility in daily life. However, IoT is not limited to private consumption; its deployment in industrial settings is also redefining manufacturing, planning, and energy distribution realms. The Industrial IoT (IIoT), typified by sensors and automation, is aimed at enhancing operational effectiveness, predictive maintenance, and live monitoring across diverse sectors. Despite these advancements, the escalation in IoT and IIoT deployments is matched by an upsurge in cybersecurity vulnerabilities, such as IoT malware. These threats bear significant implications, with the potential to disrupt not only consumer devices but also vital industrial operations. A security breach could have dire outcomes, ranging from personal data exposure to the crippling of fundamental infrastructure, even escalating to widespread, coordinated attacks on crucial systems.

IoT malware presents distinct challenges that stem from the heterogeneity and widespread presence of devices, from household gadgets to advanced industrial controls. The design of many IoT devices, especially those in industrial contexts, emphasizes utility over security, rendering them susceptible to assorted cyber threats. For example, the infamous IoT Botnet Malware Mirai heavily relies on the default system password of these Linux-based IoT devices for self-propagation. In F. Meneghello et al.’s [1] study, the authors mentioned that many IoT products do not support strong security mechanisms and can be easily targeted. Kenneth Kimani et al. [2] also addressed security challenges and their severity for the smart grid, which is a power system with IoT devices. According to NBC News [3], in 2009, an electrical facility in Puerto Rico may have lost hundreds of millions of dollars due to the crafty hacking of smart home electrical meters. In 2022, a Ukrainian critical infrastructure organization was targeted, and substations were attacked, causing a massive power outage [4]. Thus, the detection of IoT malware needs to be addressed immediately. Robust, efficient detection methods are essential to ensure the integrity, confidentiality, and availability of IoT infrastructures.

In tackling such security concerns, the application of anomaly detection, which is a type unsupervised learning, stands out as a feasible technique. Contrary to conventional training on labeled datasets, unsupervised learning utilizes the vast amounts of unlabeled network data generated by IoT systems in their normal operation. This strategy affords several benefits in the IoT sphere. Primarily, the immense and diverse data generated by innumerable devices are not labeled, while labeling these data for supervised learning can be slow and resource intensive. Additionally, IoT’s dynamic environment and evolving malware methodologies quickly outdate static, labeled datasets. In contrast, unsupervised learning can nimbly accommodate shifts and trends within real-time data streams, offering a more robust and adaptive solution for malware detection.

In this study, we examine the nuances of IoT malware data and the function of detection frameworks in protecting IoT devices. We evaluate the pros and cons for unsupervised learning techniques and illustrate their potential in meeting the complex requirements of IoT security in a world of growing connectivity. We aim to contribute to this field of study as follows:

- The Term Frequency–Inverse Document Frequency (TF-IDF) method is used to transform categorical features that cannot be directly encoded into numerical features, the values of which are random patterns with a limited set of letters or characters. This method is significantly better than directly encoding or selecting the most frequent patterns after one-hot encoding because it does not need to remove any features, risking information loss, and it can restrict the dimensions of the data to an acceptable range. This method also has the potential to be combined with n-grams to extract more useful patterns.
- We compared the anomaly detection results of one-class classification with multi-class classification. One-class classification trains the model with only benign NetFlow samples to achieve 100% recall with a reasonable level of precision. We achieved 100% recall with above 80% precision across all test data sets using Isolation Forest and 100% recall with above 90% precision across all test data sets using deep autoencoder.

The remainder of this paper is organized as follows. In Section 2, we review related works on malware detection using different methods, specifically from supervised learning to unsupervised learning and one-class classification. Section 3 introduces the details of the dataset that we used in this study. Section 4 discusses the methodology used in this study, including the data processing and feature extraction methods and anomaly detection algorithms of one-class SVM, Isolation Forest, and deep autoencoder. Evaluation metrics are also introduced in Section 4. The results and comparisons are discussed in Section 5. Finally, Section 6 concludes the study and explores future works.

## 2. Related Work

### 2.1. Supervised Learning

IoT security has gained significant attention in recent years, with numerous studies focusing on malware detection using various machine learning techniques. However, traditional approaches have predominantly relied on supervised learning methods for high accuracy because it is much easier to access a public dataset, which is often labeled. For studies that generate their own data, the size is generally small, so it will not be expensive to label. Pajouh et al. [5] proposed a supervised machine learning model, showcasing the efficiency of supervised learning algorithms in detecting malware with high accuracy but also pointing towards challenges in handling evolving malware threats. Although they have achieved decent results, with 96% detection accuracy (precision) and a 4% false positive rate, they used a relatively small dataset which contains 152 malware and 450 benign and then they used SMOTE to up-sample the data size to double, triple, and quintuple. Sudheera et al. [6] used an exceptionally large dataset that contains multiple scenarios; their goal was to detect and identify the attack stages in IoT networks. They also used supervised learning algorithms such as SVM, KNN, and RF. They achieved 99% accuracy in four datasets that are different combinations of scenarios present in the large dataset.

Deep learning has also been used in previous studies, and different frameworks have been proposed to improve the performance of malware detection. Deep learning has multiple advantages over traditional machine learning that include deep features, continuous learning, and the possibility of handling more complex and customized structures. Sahu et al. [7] proposed a hybrid model that uses CNN to extract high-level features and then classify using LSTM; they reached 96% accuracy (97% recall on malware) for all types of attacks in the dataset. There are also studies that use deep learning on images representations of malware like those by Cui et al. [8] and Vinayakumar et al. [9]. The later study also addressed the issue of biased training by using existing malware samples in the training data. They proposed a novel image processing technique to mitigate this issu

DeepAM is heterogeneous deep learning framework for intelligent malware detection created by Yanfang Ye et al. [10], which explores a deep learning architecture for malware detection using autoencoders and multilayer restricted Boltzmann machines, showing improvement over traditional methods. Although they used an autoencoder, which can be used as an anomaly detection deep learning structure, they used it as a feature compression layer instead of using it as an unsupervised anomaly detection tool.

### 2.2. Unsupervised Learning

In real world scenarios, labeled data would require extra effort and cost, which are often not applicable. Fang et al. [11] discussed the vulnerability of models based on supervised learning to specific attacks, presenting a reinforcement learning framework to evade anti-malware engines, highlighting the limitations of supervised learning in adapting to sophisticated malware modifications. Jahromi et al. [12] demonstrate an enhanced LSTM method for malware threat hunting, leveraging supervised learning for accurate detection, yet emphasizing the need for robust methods against new malware variants. A knowledge transfer-based semi-supervised federated learning framework for IoT malware detection is explored by Pei et al. [13], indicating the potential of combining supervised and unsupervised learning to mitigate data labeling challenges. However, semi-supervised learning also requires a portion of labeled data.

In Pu et al.’s [14] study, the authors presented an unsupervised anomaly detection method that combines sub-space clustering and a one-class SVM to detect attacks without any prior knowledge, and they stated that they achieved better performance for existing techniques. Zhang et al. [15] proposed a novel unsupervised model based on adversarial autoencoder and deep clustering; they utilized deep clustering to preserve the loss of the dynamic encoder reconstruction and improved the results by 42.2% based on traditional PCA feature extraction methods. However, these clustering methods are based on a pre-defined threshold that would still require using malware and benign samples in the training set and rely on the labels which are not used in training to define the threshold.

### 2.3. One-Class Classification

Acquiring malware samples, especially real-world malware samples, could also be challenging. So, a study of the feasibility of one-class classification, which only requires unlabeled benign samples, could also be useful. Tajoddin et al. [16] presented RAMD, a registry-based anomaly malware detection method using one-class ensemble classifiers. Mahmoud Al-Qudah et al. [17] presented an enhanced classification model based on a one-class SVM (OCSVM) classifier for detecting deviations from normal memory dump file patterns as malware. It integrates OCSVM and PCA for increased sensitivity and efficiency.

## 3. Dataset

The dataset used in this project is called IoT-23 [18]. This dataset was released in January 2020 and compiled by the Stratosphere Laboratory at CTU University in the Czech Republic and funded by Avast Software. This dataset comprises 23 different network traffic captures (scenarios); 20 are from IoT devices infected with malware, each labeled with the malware name, and 3 are from benign IoT devices including a Philips HUE smart lamp, an Amazon Echo, and a Somfy smart door lock. These captures were made on real hardware devices in a controlled environment with normal internet access. The dataset includes detailed information such as README.md files, original pcap files, Zeek conn.log.labeled files with network flow and behavior labels, and other analysis files. The coon.log.labeled files being used as datasets in this study are labeled NetFlows generated by Zeek based on the pcap files for each individual scenario. Different than network traffic in the pcap files, NetFlow is a network protocol developed by Cisco for collecting IP traffic information and monitoring network flow. Cisco standard NetFlow version 5 defines a flow as a unidirectional sequence of packets that all share seven values which define a unique key for the flow. The seven attributes are as follows: ingress interface (SNMP ifIndex), source IP address, destination IP address, IP protocol number, source port for UDP or TCP (0 for other protocols), destination port for UDP or TCP (type and code for ICMP, or 0 for other protocols), and IP Type of Service.

## 4. Methodology

**Figure 1:**
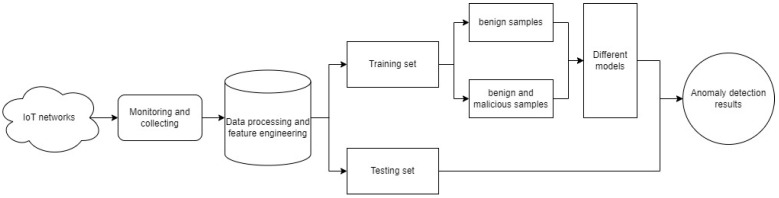
The process of the anomaly detection experiment.

Figure 1 shows the process of our method. In this study, our method is divided into 2 main parts: the first part is data processing and feature engineering, and the second part is anomaly detection. In the first part, we focus on cleaning the data and extracting useful features from the data while converting the categorical features into numerical features by applying encoding techniques TF-IDF and one-hot encoding.

For anomaly detection, most previous studies have traditionally relied on supervised learning methods, necessitating well-labeled datasets for training to build hybrid models for static or dynamic analysis. This conventional approach, while beneficial in structured environments, encounters significant challenges in the IoT context, where labeling vast amounts of data is impractical due to the sheer volume and heterogeneity. Only a limited number of studies have ventured into exploring the use of unlabeled data, which this project aims to address, because there remains a significant research void in comprehensively employing these unsupervised methods for IoT malware detection that becomes more important given the practical difficulties in obtaining labeled data in real-world settings.

### 4.1. Data Processing and Feature Engineering

The data from both captures are merged and processed together to ensure consistency across all features. Distinct labels are assigned to samples from different captures to facilitate easy separation later. The conn.log.labeled file is treated as a CSV file containing 21 features, with 20 features listed in Table 1 and the last feature being the detailed label. In this project, the original detailed label, which specifies the type of malware attack for each sample, is simplified to a binary label indicating whether it is benign or malicious. Consequently, the detailed label is not used. Features such as the timestamp and UID, which do not contribute to the analysis, are removed. Additionally, features local_orig and local_resp, which are entirely empty, and the services feature, which contains over 99% missing values, are also removed. This leaves 15 features, comprising 7 numeric and 8 categorical features. The categorical features are encoded into numeric values to facilitate processing by the algorithm.

The remaining categorical features, excluding connection state and connection history, are IP addresses and port numbers. Port numbers are categorized into three labels: well-known ports (0-1023), registered ports (1024-49,151), and dynamic/private ports (49,152-65,535). For IP addresses, given the extremely low appearance of IPv6 addresses in the datasets used, each address is split into four separate numeric features, and IPv6 addresses are split into four zeros. This approach is supported by Shao et al.’s study [19], which found that splitting IPv4 addresses into four numeric features performs better than one-hot encoding. Unlike their study, which dealt with a limited number of different IP addresses, the dataset in this study contains a large variety of IP addresses, making one-hot encoding impractical.

In machine learning, one-hot, also called dummy variables, refers to a group of binary values among which the valid combination of values is only those with 1 s, and all the others are 0 s. One-hot encoding is a technique that is based on this representation to transform categorical features into numerical features. In this dataset, handling categorical features conn_state and history can be challenging, requiring a deeper understanding of networking and cybersecurity. For conn_state, direct one-hot encoding is feasible due to the small number of labels as Table 2 shows. Although conn_state has a limited number of labels, adding more than 10 dimensions to the dataset is not ideal, especially when some labels have very few occurrences compared to the most frequent label. Therefore, for conn_state, labels containing RST are combined into a new label RST, as they all represent an unusual reset request for the TCP connection. Similarly, labels S0 and SF are combined into one label, as they essentially represent the same condition with a minor difference in byte transformation.

A more in-depth analysis is needed for connection history, which is an extremely important feature that contains the details of the connection, and some of the details are critical in identifying malicious activities. The values of connection history are a random string pattern formed by a set of strings, as Table 3 shows, and each string represents a state in the entire connection history of each NetFlow. Thus, the values of this feature are not labels but also not numeric, and one-hot encoding cannot be applied to this feature without losing information that might be critical. We propose to use term frequency and inverse document frequency (TF-IDF), which is a technique often used in natural language processing to process this feature. Common approaches to process these types of features, such as frequency encoding or target encoding, are simple solutions; however, these approaches might not reflect the details that each value represents. By using TF-IDF, not only will it not result in too many dimensions and loss of information but also it could consider the impact of each individual string, because TF-IDF works by assigning a score to each term that reflects the importance of a term in the corpus. In this case, the corpus is the value for this feature, and the terms are each an individual string. This technique also has the potential to be combined with n-grams in a future study, when higher computational power is available, that also considers the relationship between multiple terms in a corpus. After applying this technique to the history column, it will result in 12 new features for each individual string shown in Table 3. The values for each new feature are the TF-IDF score if this string is in the original value, and all the new features are numerical.

At this stage, there are 6 labels each in conn_state and history, and 3 labels in proto, allowing for one-hot encoding to be applied. Upon a further examination of the numerical features, it is found that some features contain more than 99% of the same value, which indicates that these features are highly likely to have minimal impact on the target. To ensure in extreme cases that these types of features could also be characteristic, the frequency is calculated separately for each type of label, and if a feature contains over 99% of the same value for all types including benign, it will be removed. Consequently, the final dataset comprises 18 features.

### 4.2. Anomaly Detection Techniques

Detecting malware is essentially an anomaly detection problem; however, most previous studies have used both malicious and benign data for training, which is logical because the results will be more accurate. However, in real world situations, malicious data are not always accessible, especially for a real time malware detection system, therefore, the training must be performed only based on the normal benign data. This project delves into an underexplored territory in IoT cybersecurity research—the exclusive use of unlabeled positive data for training detection models and the aim to build on the limited existing research and advance the field by proposing methodologies that are specifically tailored to exploiting unlabeled data, ensuring both the practicality and effectiveness of malware detection in diverse IoT settings. This focus on unlabeled data represents a significant departure from traditional methods and positions our study as a crucial contribution to the evolving landscape of IoT cybersecurity.

In the scope of one-class classification, various algorithms have been utilized to identify anomalies, each with their unique strengths and weaknesses. The one-class Support Vector Machine (SVM) is a popular method that constructs a hyperplane to separate normal data from outliers, excelling in scenarios with a clear margin of separation. However, it can be computationally intensive and struggles with high-dimensional data. The Isolation Forest, on the other hand, isolates observations by randomly selecting a feature and then randomly selecting a split value between the maximum and minimum values of the selected feature. This method is particularly effective in handling high-dimensional data and is computationally efficient, but it may be less effective when the anomalies are not well-isolated or when the dataset contains noise. Autoencoders, a type of neural network architecture, are designed to reconstruct input data and identify anomalies by measuring the reconstruction error. They are highly flexible and can model complex, non-linear relationships, making them suitable for a wide range of applications. However, they require significant computational resources and substantial amounts of data for training, and their performance can be sensitive to the choice of architecture and hyperparameters. In summary, while one-class SVM is robust for well-defined margins, Isolation Forest is better for high-dimensional data, and autoencoders offer flexibility for complex patterns. Each method’s effectiveness depends on the specific characteristics and requirements of the dataset and application.

Both one-class SVM and Isolation Forest are proven to be effective for anomaly detection problems. One-Class SVM is adapted from the traditional SVM, specifically designed for anomaly detection. Isolation Forest is another unsupervised learning method for anomaly detection which is probability based. In most cases, one-class SVM has proven to have more accuracy than Isolation Forest; however, in this case, where the dataset is large and dimensions are relatively high, SVM could encounter a significant performance decrease, especially for non-linear kernels. Isolation Forest, on the other hand, handles large datasets easier and has negligible performance impact with higher dimensions.

**Figure 2:**
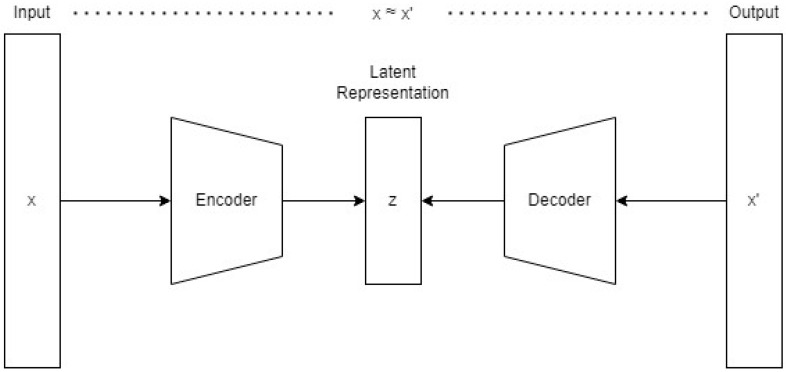
The structure of the autoencoder used in this study.

An autoencoder is a structure of deep learning anomaly detection as Figure 2 shows. It typically consists of two main parts, an encoder and a decoder. The encoder compresses the input into a latent-space representation, and the decoder reconstructs the input data from this representation. The aim is to learn a representation that captures the most notable features of the data and detect anomalies using the reconstruction error.

In this study, an autoencoder with a sequential architecture was employed for malware detection through anomaly detection. The model comprises six densely connected layers. The input layer has 17 neurons, corresponding to the dimensionality of the input data. The encoder part of the autoencoder consists of two hidden layers with 16 and 8 neurons, respectively, reducing the dimensionality of the input data to a compressed representation. The bottleneck layer, the smallest layer in the network, has 4 neurons. The decoder part mirrors the encoder, with two hidden layers of 8 and 16 neurons, respectively, aiming to reconstruct the input data from the compressed representation. The output layer has 17 neurons, matching the number of input features, and is used to compare the reconstructed data with the original input. The model has a total of 933 trainable parameters, making it a relatively lightweight neural network suitable for detecting anomalies in the context of malware detection.

In the context of determining the reconstruction error threshold to classify anomalies, using the Median Absolute Deviation (MAD) instead of percentiles to determine the threshold is a more robust approach. MAD is a measure of variability that is less sensitive to outliers in the data, making it a suitable choice for anomaly detection tasks where the goal is to identify rare or unusual observations. By calculating the MAD of the reconstruction errors between the original input and the reconstructed output of the autoencoder, a threshold can be set based on a multiple of the MAD value. This threshold can then be used to classify observations as normal or anomalous. Using MAD as the basis for the threshold helps to reduce the impact of extreme values in the data, leading to more stable and reliable anomaly detection. This approach is more robust and scalable than the percentiles method and can potentially achieve better performance for unseen data.

Median=median(X)D=|X−Median|MAD=median(D)

Let X be the dataset. The first step is to obtain the median for the data set. Then, D represents the array of absolute deviations from the median, and lastly, the MAD score is equal to the median of D. In a normal distribution, the MAD is related to the standard deviation (σ) of the equation.

MAD≈0.6745×σ

We obtain the MAD score, which is a z-score using the following equation.

$$MAD\;score=0.6745\times \frac{AD}{MAD}$$

One-class classification and anomaly detection are closely related concepts in machine learning, often used interchangeably, yet they have distinct differences. We compared the performance of one-class training with multi-class training and propose to use one-class training instead of multi-class training, because one-class training focuses on creating a model that learns to recognize instances from a single class, typically the “normal” or “benign” class, and treats any deviation from this class as an anomaly. This approach is particularly useful when there is a lack of labeled data for the “anomalous” or “malicious” class, as it allows the model to learn the characteristics of the normal class without the need for labeled examples of anomalies.

In this study, the need for one-class classification arises particularly in scenarios such as IoT malware detection, where access to labeled malicious samples is limited, and the diversity of malware types makes it challenging to cover all possible variations in a training dataset. By focusing on learning the characteristics of benign data, one-class classification provides a robust solution that can generalize well to detect not only known types of malware but also unknown variants that may emerge in the future. This ability to adapt to new threats without requiring extensive labeled data makes one-class classification a valuable approach for maintaining the security of IoT systems in a constantly evolving threat landscape.

### 4.3. Evaluation Metrics

In the context of IoT malware detection using one-class classification, the evaluation of the model’s performance is crucial to ensure its effectiveness in identifying anomalies. Commonly used metrics for this purpose are precision, recall, and F1 scores, which provide insights into the model’s accuracy and sensitivity.

Precision measures the proportion of correctly identified positive instances (true positives) out of all instances classified as positive (true positives and false positives). In the context of malware detection, a high precision indicates that the model is accurate in identifying malicious samples, with fewer benign samples being incorrectly labeled as malicious. While precision is important, it is not the sole focus in this scenario, as the cost of missing a true malware sample (false negative) can be significantly higher than the cost of a false alarm (false positive).

$$Precision=\frac{True\;Positives\;(TP)}{True\;Positives\;\left(TP\right)+False\;Positives\;(FP)}$$

Recall, also known as sensitivity or true positive rate, measures the proportion of actual positive instances (true positives) that are correctly identified by the model out of all actual positive instances (true positives and false negatives). In the context of IoT malware detection, recall is of paramount importance. A high recall means that the model can detect a considerable proportion of the malware samples, minimizing the risk of undetected threats that could compromise the security of IoT systems. Given the potentially severe consequences of missed malware detections, prioritizing recall is a strategic decision aimed at ensuring the highest level of security.

$$Recall=\frac{True\;Positives\;(TP)}{True\;Positives\;\left(TP\right)+False\;Negatives\;(FN)}$$

In practice, there is often a trade-off between precision and recall, especially in the context of anomaly detection and one-class classification. To address this trade-off, the F1 score can be used as a harmonic means of precision and recall, providing a balanced measure of the model’s performance. However, in scenarios where the primary goal is to capture as many anomalies or malware samples as possible, recall is given precedence. This means that while striving for a high recall, some decrease in precision may be acceptable to ensure that the model is sensitive to potential threats.

$$F1=2\times \frac{Precision\times Recall}{Precision+Recall}$$

In anomaly detection problems, especially in scenarios where the class distribution is imbalanced (i.e., the number of normal instances outweighs the number of anomalies), the Area Under the Receiver Operating Characteristic (AUC-ROC) curve can be a better metric than precision, recall, or the F1 score. The ROC curve plots the true positive rate (TPR) against the false positive rate (FPR) at various threshold settings, providing a comprehensive view of the model’s performance across all thresholds. This is useful in anomaly detection, where the cost of false negatives can be high. The AUC-ROC is less sensitive to class imbalance than precision, recall, or F1 score, as it evaluates the model’s ability to distinguish between classes rather than its ability to correctly label instances. Therefore, a high AUC-ROC score could indicate that the model is capable of detecting anomalies with a low rate of false positives, which is often the primary goal in anomaly detection tasks.

In addition to the above metrics, the Precision–Recall (PR) curve is another valuable tool for evaluating the performance of a malware detection model, especially in the context of imbalanced datasets. The PR curve plots the precision (y-axis) against the recall (x-axis) at various threshold settings, providing a detailed view of the trade-off between precision and recall for different threshold values. This perfectly fits the goal of achieving higher recall in this study. Unlike the ROC curve, which plots the true positive rate against the false positive rate, the PR curve is more informative for imbalanced datasets where the number of negative instances (benign samples) significantly outweighs the number of positive instances (malware samples).

In scenarios where the actual number of negatives is large, a model might produce a large number of false positives while still maintaining a small false positive rate (FPR), leading to a misleadingly high Area Under the ROC Curve (AUC-ROC) score. This can create a false sense of security, as the model may not be as effective as the AUC-ROC score suggests. In contrast, the Precision–Recall curve is more sensitive to the number of false positives, as precision directly incorporates the number of false positives into its calculation. Therefore, a drop in precision due to an increase in false positives is immediately visible in the PR curve.

For this reason, in the context of IoT malware detection, where the cost of false negatives is high and the dataset is often imbalanced for anomaly detection problems, the PR curve can provide a more realistic assessment of the model’s performance. A high area under the Precision–Recall curve (AUC-PR) indicates that the model could achieve high precision while maintaining high recall, which is crucial for effectively detecting malware without overwhelming the system with false alarms. Furthermore, the Precision–Recall curve does not only provide a more realistic performance evaluation. The area under the PR curve (AUC-PR) can also be a more informative summary statistic than the traditional AUC.

## 5. Results and Comparison

In this section, we primarily focus on comparing the performance of multi-class training, which utilizes both benign and malicious unlabeled samples, with that of one-class training, which exclusively employs benign samples in the training dataset. We evaluate two distinct algorithms, Isolation Forest and deep autoencoder, across both training approaches. While one-class SVM is theoretically capable of achieving superior results in anomaly detection tasks, its practical application is limited by its inability to efficiently handle large-scale datasets. This limitation is particularly pronounced for non-linear SVMs, rendering them impractical for datasets of the size considered in this study.

### 5.1. Train-Test Split

For the benign data, we allocate 40% for training, 30% for testing, 20% for validation, and the remaining 10% for tuning. In the one-class training scenario, the entire 40% of the benign data is used as the training set. However, for multi-class training, we modify the training set by replacing 20% of the benign samples with malicious samples to achieve an 80:20 ratio between benign and malicious data. The test sets for both training approaches maintain an 80:20 ratio of benign to malicious samples. We select three major attack types that have a total of over 99% among all malicious samples. For each of the three major attack types—PartOfAHorizontalPortScan, DDoS, and Okiru—we select an equal number of samples corresponding to 20% of the benign test samples from each attack type, resulting in four test sets for each attack category. In the rest of the attack types, there are total of 4981 samples combined after removing duplicates; these samples are combined into one separate test set called attack, and a small benign sample is selected to also create an 80:20 ratio for this test set. In a nutshell, we have 15,334,261 samples for all the training sets and 13,494,149 samples for all the test sets.

### 5.2. Model Tuning

The performance of anomaly detection models, such as Isolation Forest and autoencoders, is significantly influenced by the choice of hyperparameters. For Isolation Forest, key hyperparameters include the number of trees and the sample size used for building each tree. Increasing the number of trees generally improves the model’s robustness and stability by averaging out the anomalies, but it also increases computational cost. Similarly, a larger sample size can capture more data variability, enhancing the model’s accuracy, yet it may also lead to higher memory consumption and longer training times. On the other hand, autoencoders have several critical hyperparameters, including the architecture, learning rate, and regularization techniques. A different technique involving a deeper or wider network can capture more complex patterns but may also risk overfitting, particularly with limited data. The learning rate affects how quickly the model converges; too high a learning rate can lead to instability, while too low can slow down training. Regularization techniques like dropout or L2 regularization help prevent overfitting by adding noise or penalizing large weights, respectively. The proper tuning of these hyperparameters is essential for achieving optimal performance, as it balances the trade-off between model complexity, computational efficiency, and generalization capability. Consequently, hyperparameter optimization, often through techniques like grid search or Bayesian optimization, is a crucial step in deploying effective anomaly detection models.

However, the influence of hyperparameters is not always as expected; proper tests are still needed. In this study, because we have already prepared tunning sets with the same distribution as the training and testing sets and proper feature engineering, we do not need to consider the random selection of max samples and max features. This left the Isolation Forest model with 2 main hyperparameters, which are contaminations and the number of estimators. We conducted a grid search on 3 values for each of the hyperparameters, which are 0.05, 0.1, and 0.15 for contamination and 100, 200, and 300 for the number of estimators, with a total of 9 combinations. As expected, the number estimators have a direct impact on the training time; the increase in time is approximately the same as the ratio of increasing the number of estimators. However, increasing the number of estimators does not directly lead to higher accuracy; in some cases, it even reduced the accuracy, but overall, these 3 different numbers of estimators achieved approximately the same results. Considering the performance, 100 estimators were selected. Contamination is the anomaly threshold for the Isolation Forest model, and for one-class classification problems, even though the training sets only contain normal data, a proper threshold can still be helpful to rule out a small percentage of outliers to make the model more sensitive to normal data. The precision and recall trade off needs to be considered when tuning this parameter. Based on the results on the tuning samples, 0.15 contamination produces lower accuracy than 0.05 and 0.1, which both reached a 99% F1 score on the tuning sets with very close results. Because Isolation Forest models can handle large datasets with good performance, we trained two models using both 0.05 and 0.1 contamination, and 0.1 achieved better results than 0.05.

For the autoencoder, we applied early stopping for the number epochs that stops training if the validation loss does not improve more than 0.0001 over 5 epochs and a learning rate schedule starting at 0.001 learning rate with a 0.9 decay rate. Further, for the activation function, because the output layer is between 0 and 1, we choose the sigmoid as the activation function for the output layer. Then, we experimented with activation function ReLU, leaky ReLU, and Tanh for a 4-layer architecture with 8 and 4 as the dimensions. ReLU achieved the better results. Then, we experimented with different batch sizes 64, 128, and 256. Technically, smaller batch size can help generalize better by introducing noise in the gradients, with slower training time. Further, as expected, batch size 64 achieved better results and the training time slowed down at approximately the same rate at which the batch size increased, but using batch size 64, the model still converged within 20 epochs, which is an acceptable performance. Moreover, we have also added drop out layers and L2 regularization, but adding these layers does not improve the results, and we presume that this is because of the low complexity of the data. Finally, we experimented with a 6-layer architecture with 16, 8, and 4 as dimensions, and it achieved close but better results on the tuning sets over the 4-layer architecture. As for the threshold, in Section 4.2, we introduced using a z-score to determine the threshold for the reconstruction error produced by the autoencoder, which is much more robust compared to a percentile threshold like the contamination value in the Isolation Forest model. For the cut-off value, which represents the number of standard deviations away from the mean, while calculating the z-score, 3 to 3.5 is a common range used as the cut-off value. A high cut-off value could decrease the recall; however, we reached 100% recall within this range, so we increased the cut-off, and it reached the highest precision while maintaining 100% recall.

### 5.3. Isolation Forest

One-class SVM could potentially achieve better performance for anomaly detection problems over Isolation Forest. However, the speed makes it intolerable when dealing with a large data set, especially for non-linear kernels. After reducing the dimensions, the size of the data set is still too big, even for linear SVM. On the other hand, Isolation Forest is an algorithm specifically designed for anomaly detection. It is based on the principle of isolating anomalies using decision trees, which are constructed by randomly selecting features and splitting values. This approach allows Isolation Forest to handle large datasets efficiently, as the computational complexity is linear with the number of samples and logarithmic with the number of trees in the forest. Additionally, Isolation Forest is less sensitive to the dimensionality of the data, as each tree considers only a subset of features at each split. This characteristic enables Isolation Forest to maintain fast performance and produce decent results even when the dimensionality of the data is high, making it a more suitable choice for anomaly detection in large and high-dimensional datasets. The following table shows the precision, recall, F1 scores, and time-related performance for all three test sets with two different training strategies.

In Table 4, the results using one-class training are significantly better than those using multi-class training, which means the training set contains both benign and malicious samples. Using the one-class training method, both the DDoS test set and Okiru test set achieve 100% recall with 84% precision, which means that all the anomalies are captured. The PartOfHorizontalPortScan test set also achieves 95% recall, with 81% precision, compared to the results using multi-class training, where only the Okiru test set reaches 100% recall but with only 54% precision. The DDoS and PartOfHorizontalPortScan test sets achieve 73% and 76% recall, with 46% and 47% precision. The training times and testing times are also slightly faster when using one-class training compared to using multi-class training. The results prove that using one-class training is not only feasible but also achieves much better results and are also more robust to several types of malware attack.

**Figure 3:**
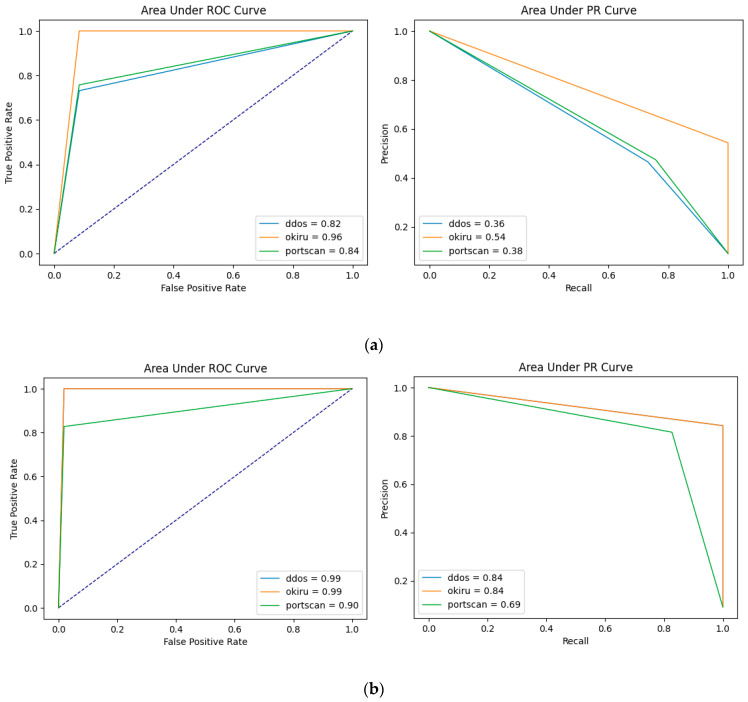
(**a**) AUC-PR curve and AUC-ROC curve for multi-class training using isolation forest. (**b**) AUC-PR curve and AUC-ROC curve for one-class training using isolation forest.

Because the test sets are imbalanced, precision, recall scores may not accurately represent the performance of the models. Figure 3 shows the AUC-ROC and AUC-PR curves for all the test cases. It is clear to see that in both AUC plots, the area under curves for one-class training are bigger than the multi-class training, which means the overall performance is better. Further, the gaps between curves are also smaller when using one-class training, and the curves for DDoS and Okiru test sets are even overlapped, which proves the robustness of the model.

### 5.4. Deep Learning Autoencoder

In previous tests for Isolation Forests, it has already been proven that one-class training with only benign samples is feasible and provides better results over multi-class training. However, not all test sets achieved 100% recall, and the precisions also have room to improve. Deep learning autoencoders can achieve better performance for anomaly detection compared to Isolation Forest, especially when dealing with large and complex datasets. Autoencoders can learn non-linear and high-dimensional representations, making them particularly effective for anomaly detection, as they can capture the underlying structure of normal data and identify deviations or anomalies more accurately. Furthermore, autoencoders can scale well with large datasets due to the parallel processing capabilities of modern deep learning frameworks and hardware accelerators like GPUs. In contrast, while Isolation Forest is efficient and effective for many anomaly detection tasks, it may not capture the complex dependencies and patterns in the data as well as autoencoders, particularly in high-dimensional spaces. Table 5 shows the performance metrics for 3 test sets using the same one-class training set.

**Figure 4:**
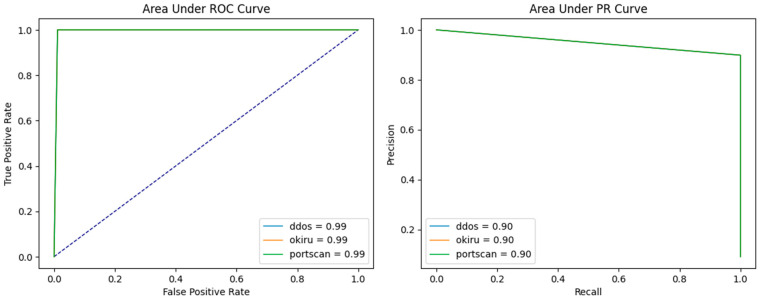
AUC-PR curve and AUC-ROC curve for one-class training using autoencoder.

As Table 5 shows, all 3 test sets achieved the same results, which are 100% recall and 90% precision using one-class training compared to an average 77% recall and 96% precision using multi-class training, which meets the expectation that adding malicious samples to the training set will increase the model’s sensitivity to benign samples but may decrease the model’s sensitivity towards malicious samples and robustness. Compared to the isolation forest model, not only did the performance improve but also it achieved perfect robustness over three different types of attacks. Speed-wise, the autoencoder was trained for 20 epochs, but it stopped converging at 17 epochs, and it took 5 m 46 s for each epoch. Considering that the training is not designed to be performed on any IoT devices and accounts for the improvement in performance, the longer training time is acceptable. The testing times are also longer than using Isolation Forest; for each sample, it takes an average 41.13 µs. compared to 23.11 µs using the Isolation Forest model. Figure 4 below is the AUC plots, which also indicate that the area is larger than the Isolation Forest plots, and all three curves are perfectly aligned, indicating the robustness of the model.

**Figure 5:**
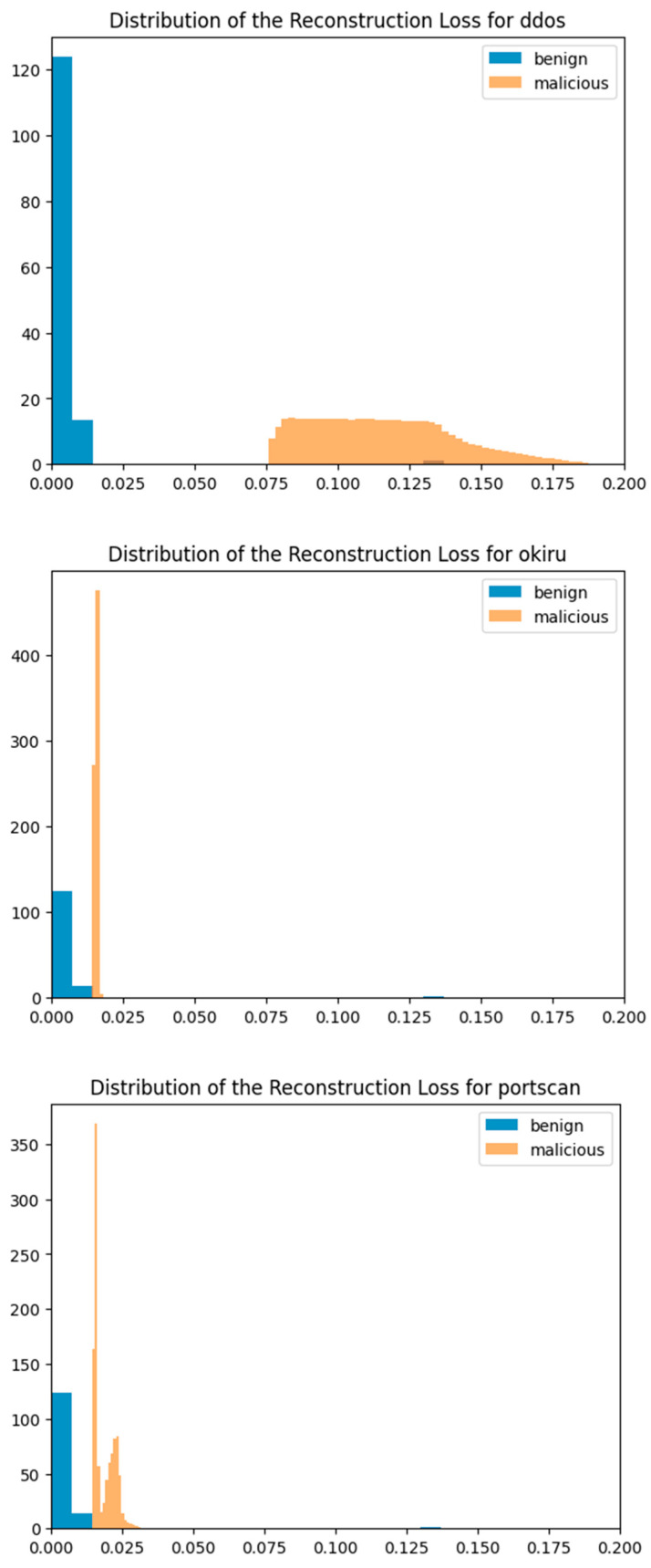
Reconstruction MSE distribution for benign and malicious classes on three testing sets.

Because the autoencoder detects anomalies by using the reconstruction error, it could also be helpful to visualize the distribution of the errors. Figure 5 below displays the distribution of reconstruction loss for all three test cases. The reconstruction loss for benign and malicious samples are well separated. In the Okiru and PartOfHorizontalPortScan test sets, there are overlapping situations for benign and malicious samples. Furthermore, for all three test cases, there is a small portion of benign samples that fall between 0.125 and 0.150 on the x-axis, which will be misclassified as false positives.

## 6. Discussion

Scalability is a critical factor when evaluating anomaly detection models, particularly in the context of the increasing complexity and volume of IoT data. Isolation Forest is inherently scalable due to its tree-based structure, which allows it to handle large, high-dimensional datasets efficiently. Its linear time complexity with respect to the number of samples makes it well-suited for real-time anomaly detection in IoT environments, where data streams continuously and unpredictably. Additionally, Isolation Forest’s ability to operate effectively with minimal tuning further enhances its scalability across diverse IoT applications. Conversely, autoencoders, which are neural network-based models, offer robust capabilities to capture complex, non-linear patterns in data. However, their scalability is constrained by several factors. Training autoencoders on large datasets can be computationally intensive and time-consuming, often requiring significant computational resources such as GPUs or distributed computing frameworks. Moreover, the performance of autoencoders heavily relies on architecture design and hyperparameter tuning, which can be challenging and resource demanding as data complexity increases. Despite these challenges, autoencoders’ flexibility in modeling intricate data relationships makes them valuable for specific IoT applications where detailed anomaly characterization is crucial. Furthermore, the computation problem can be solved by server-based training, especially in IoT systems, where data need to be collected from all the IoT devices instead of just one device; this makes it more practical and feasible to utilize server-based workflows for data processing and model training, and the trained model will be deployed to the IoT devices to monitor the network and detect malware. As for the testing speed, even though the Isolation Forest model still tests faster than the anomaly detection model, as Section 5 shows, both models can test one sample within one millisecond, making the fast testing speed advantage of the Isolation Forest model more negligible. In summary, while Isolation Forest provides a scalable and efficient solution for anomaly detection in large-scale IoT data, the importance of these advantages diminished for server-based training, while autoencoders offer powerful capabilities for capturing complex data patterns.

When using machine learning models for malware detection, interpretability is a crucial factor for cybersecurity experts. Isolation Forest, being an ensemble method, works by recursively partitioning data points and is relatively interpretable because it provides insights into anomalies by identifying data points that require fewer partitions to isolate. This allows experts to understand why certain points are classified as outliers, aiding in the investigation of potential malware. On the other hand, autoencoder models, which are a type of neural network, encode data into a lower-dimensional space and then reconstruct it, flagging significant reconstruction errors as anomalies. While effective, the interpretability of autoencoders is generally lower due to their complex, black-box nature. Techniques such as the visualization of latent spaces, reconstruction error analysis as in Figure 5, and layer-wise relevance propagation can help experts gain insights into the model’s decision-making process. Balancing detection performance with interpretability is essential, as it empowers cybersecurity experts to trust and effectively act on the models’ outputs in the fight against malware. However, these interpretations are still based on the models’ outputs, which requires the cybersecurity experts to have knowledge on machine learning to be able to understand. The employment of explainable AI (XAI) can help with this situation. XAI aims to make machine learning models more transparent and understandable by providing explanations of how decisions are made. For instance, in the context of Isolation Forest, XAI methods could visualize which features contribute the most to the isolation of a data point, effectively showing decision weights and helping experts understand which aspects of the data are most indicative of anomalies. Similarly, for autoencoders, XAI tools can help by identifying which features or input dimensions have the largest reconstruction errors, thus highlighting the parts of the data that the model finds suspicious. By presenting these insights in an accessible way, XAI can bridge the gap between complex model outputs and the practical understanding needed by cybersecurity experts to make informed decisions. Incorporating XAI can significantly enhance the usability of these models in real-world cybersecurity applications by making the detection process more transparent and the results more actionable for experts without deep machine learning expertise.

## 7. Conclusions and Future Work

The findings from our research suggest a significant advancement in the domain of malware detection, favoring a one-class training approach over a multi-class training approach. Our experiments reveal that one-class training is not only feasible but also yields superior results compared to its multi-class counterpart. This success is primarily due to the one-class approach’s inherent capacity to mitigate the training bias associated with specific types of malware data. Such biases often skew the learning process and compromise the model’s generalizability. Additionally, one-class training offers a more economical and streamlined method for detecting malware by obviating the need for extensive malicious data collection and exhaustive labeling efforts. The robust performance of the one-class models, despite the reduced requirement for labeled data, marks a promising direction for efficient and cost-effective malware detection strategies.

Future research endeavors will concentrate on refining the data foundation, feature representation, and model architecture to bolster the effectiveness of malware detection systems further. The next phase will seek to incorporate data that mirrors the unpredictability and complexity of real-world scenarios more closely, thus addressing the limitations of data collected from controlled environments. Emphasis will also be placed on enhancing feature extraction techniques, particularly using n-grams within connection history analysis. This shift aims to capture a broader spectrum of behavioral patterns, though it may result in an increased number of features. Therefore, feature selection and reduction techniques will be essential to manage the expanded feature set without compromising the model’s interpretability or performance.

In parallel, efforts will be made to explore the potential of more intricate autoencoder designs. The relatively basic autoencoder employed in this study offers a foundational starting point, suggesting a vast unexplored potential for complex architectures. Innovations in autoencoder structures, potentially integrating advanced neural network techniques, hold the promise of significantly improving the model’s capability to detect subtle and sophisticated patterns indicative of malware presence.

## Figures and Tables

**Table 1:** All features in the original dataset.

Feature	Description	Process
ts	Timestamp of when the connection was logged.	Removed.
uid	Unique identifier for the connection.	Removed.
id.orig_h	Originating host’s IP address.	Split to 4 new features for each part of the IP.
id.orig_p	Originating host’s port number.	Create 3 new labels based on the range of port number and one-hot encoded.
id.resp_h	Responding host’s IP address.	Split to 4 new features for each part of the IP.
id.resp_p	Responding host’s port number.	Create 3 new labels based on the range of port number and one-hot encoded.
proto	Protocol used for the connection.	One-hot encoded.
service	Service being accessed, if detectable.	Removed due to high missing values.
duration	Duration of the connection in seconds.	Removed due to low variance.
orig_bytes	Number of bytes sent by the originating host.	Removed due to low variance.
resp_bytes	Number of bytes sent by the responding host.	Removed due to low variance.
conn_state	State of the connection at the time of logging.	Combined label S1, S2, and S3 to S and one-hot encoded.
local_orig	Boolean indicating if the originating host is part of the local network.	Removed due to high missing values.
local_resp	Boolean indicating if the responding host is part of the local network.	Removed due to high missing values.
missed_bytes	Number of bytes missed in the connection due to dropped packets.	Removed due to low variance.
history	Sequence of connection state history.	Encoded using TF-IDF.
orig_pkts	Number of packets sent by the originating host.	Unchanged.
orig_ip_bytes	Number of IP layer bytes sent by the originating host.	Unchanged.
resp_pkts	Number of packets sent by the responding host.	Removed due to low variance.
resp_ip_bytes	Number of IP layer bytes sent by the responding host.	Removed.
label	Label indicating whether the connection was benign or the type of malicious.	Changed to binary.

**Table 2:** All possible values for the connection state feature.

Conn_State	Summarized State	Process
S0	Connection attempt seen, no reply	Unchanged.
S1	Connection established, not terminated (0 byte counts)	Combined to new label S.
SF	Normal establish and termination (>0 byte counts)	Merged into S0.
REJ	Connection attempt rejected	Unchanged.
S2	Established, Orig attempts close, no reply from Resp	Combined to new label S.
S3	Established, Resp attempts close, no reply from Orig	Combined to new label S.
RSTO	Established, Orig aborted (RST)	Combined to new label RST.
RSTR	Established, Resp aborted (RST)	Combined to new label RST.
RSTOS0	Orig sent SYN then RST; no Resp SYN-ACK	Combined to new label RST.
RSTRH	Orig sent SYN-ACK then RST; no Orig SYN	Combined to new label RST.
SH	Orig sent SYN then FIN; no Resp SYN-ACK (“half-open”)	Unchanged.
SHR	Resp sent SYN-ACK then FIN; no Orig SYN	Unchanged.
OTH	No SYN, not closed. Midstream traffic. Partial connection.	Unchanged.

**Table 3:** All letters and characters that compose the string pattern in the history feature.

History	Description
S	A SYN without the ACK bit set
H	A SYN-ACK (“handshake”)
A	A pure ACK
D	Packet with payload (“data”)
F	Packet with FIN bit set
R	Packet with RST bit set
C	Packet with a bad checksum
I	Inconsistent packet (Both SYN and RST)
Q	Multi-flag packet (SYN and FIN or SYN + RST)
T	Retransmitted packet
W	Packet with zero window advertisement
^	Flipped connection

**Table 4:** Comparing the scores for multi-class training with one-class training using Isolation Forest.

Training Method	Training Time	Testing Set	Test Time	Precision	Recall	F1
multi-class training	8 m 51.5	ddos	455.5 s	0.46	0.73	0.57
		okiru	414.4 s	0.54	1	0.7
		portscan	395.6 s	0.47	0.76	0.58
		average	421.8 s	0.49	0.83	0.62
one-class training	6 m 6.1 s	ddos	311.1 s	0.84	1	0.91
		okiru	311.9 s	0.84	1	0.91
		portscan	308.5 s	0.81	0.83	0.82
		average	310.5 s	0.83	0.94	0.88

**Table 5:** Scores for one-class training using autoencoder.

Training Method	Training Time	Testing Set	Test Time	Precision	Recall	F1
multi-class training	5 m 46 s * 17 epochs	ddos	321 s	0.88	0.76	0.82
		okiru	321 s	1	0.78	0.88
		port	322 s	1	0.78	0.88
		average	321.3 s	0.96	0.77	0.86
one-class training	11 m 52 s * 8 epochs	ddos	538 s	0.9	1	0.95
		okiru	555 s	0.9	1	0.95
		port	553 s	0.9	1	0.95
		average	548.7 s	0.9	1	0.95

The * means the average training time per epoch “times” the total number of epochs. It can be changed to the total training time followed by parentheses with the average time. For example, 60 m 2 s (5 m 46 s/epoch).

## Data Availability

Data is available upon request.
